# Selective 6H-SiC White Light Emission by Picosecond Laser Direct Writing

**DOI:** 10.1038/s41598-017-18685-0

**Published:** 2018-01-10

**Authors:** Sicong Wang, Lingfei Ji, Lin Li, Yan Wu, Yongzhe Zhang, Zhenyuan Lin

**Affiliations:** 1 Institute of Laser Engineering, Beijing University of Technology, Beijing, 100124 China; 2 Laser Processing Research Centre, School of Mechanical, Aerospace and Civil Engineering, The University of Manchester, Manchester, M13 9PL UK; 3 College of Material Science and Engineering, Beijing University of Technology, Beijing, 100124 China

## Abstract

Displaying a full or tuneable emission spectrum with highly efficient is significant for luminescent materials used in solid-state lighting. Silicon carbide (SiC) has potential for use in photoelectric devices that operate under extreme conditions. In this paper, we present a method to selectively modify the photoluminescence (PL) properties of SiC by ultrafast laser direct writing. Based on this method, visible white PL could be observed by the naked eye at room temperature under ultraviolet excitation. By increasing the laser power intensity from 40 to 80 MW/cm^2^, the PL of the irradiated samples increased and pure white sunlight-like emission with controlled colour temperature was realised. The optimised laser power intensity of 65 MW/cm^2^ achieved a desirable colour temperature similar to that of sunlight (*x* = 0.33, *y* = 0.33 and colour temperature of 5500 K) and suppressed blue emission. By direct laser irradiation along designed scanning path, a large-scale and arbitrary pattern white emission was fabricated. The origin of the white luminescence was a mixture of multiple luminescent transitions of oxygen-related centres that turned the Si-C system into silicon oxycarbide. This work sheds light on new luminescent materials and a preparation technique for next-generation lighting devices.

## Introduction

An ideal solid-state light source is expected to be highly efficient and reliable, suitable for integration and display a full or tuneable emission spectrum^1–3^. Novel semiconductor materials^4–11^, including ZnO, ZnS, and Si-based oxycarbide or oxynitride, have been investigated as luminescent substances. Their material and device structures have been manipulated to optimise performance. Largely owing to the achievement of highly efficient group III nitride-based blue light-emitting diodes (LEDs), white LEDs that are based on blue LED excitation of yellow phosphor coatings that contain rare-earth elements are now commonly used for lighting applications. However, such an emission spectrum composition with excess blue component leads to a low colour-rendering index of up to 68 and high colour temperature of 7400 K, increasing the risk of ‘blue hazard’. To this end, attention is increasingly turning to the study of ultraviolet (UV)-excited non-rare-earth fluorescent materials.

SiC is a candidate for white light emission because of its wide band gap and capability of being doped to achieve various radiative transitions in the visible spectrum. In addition, SiC is an ideal semiconductor material for high-frequency, -power, -temperature and -radiation microelectronic applications because it has high breakdown threshold, thermal conductivity and saturation velocity^12,13^. However, the indirect band gap of SiC means that its luminescence intensity is not strong enough for applications such as lighting and display devices. Thus, a current challenge is to tailor the luminescence properties of SiC.

Here, we demonstrate an approach to modify SiC surfaces to realise white light emission using an ultrafast laser. A picosecond pulsed laser (10-ps duration) with a peak power intensity of the order of 10^12^ W/cm^2^ is used to modify the surface state of n-type 6H-SiC in air at room temperature, which induces visible white photoluminescence (PL) under UV excitation. The laser modification process is controlled through laser parameters following numerical simulation of the time dependence of injected energy to estimate its heat flow and oxidation features. Visible white PL with desirable colour temperature similar to that of pure white sunlight (*x* = 0.33, *y* = 0.33 and colour temperature = 5500 K) is observed by the naked eye at room temperature under UV excitation. By direct laser irradiation along a designed scanning path, a large-scale, arbitrary pattern displaying selective visible white light emission is fabricated. By characterising the laser-modified surface, the origin of the white luminescence is found to be related to the combination of several Si-O luminescent centres. Laser annealing of the processed materials is conducted to study the mechanism of their defect-derived luminescent properties. A laser-fabricated p-n junction is also produced, showing the potential to fabricate semiconductor devices with rectifying properties.

## Results and Discussion

**Figure 1:**
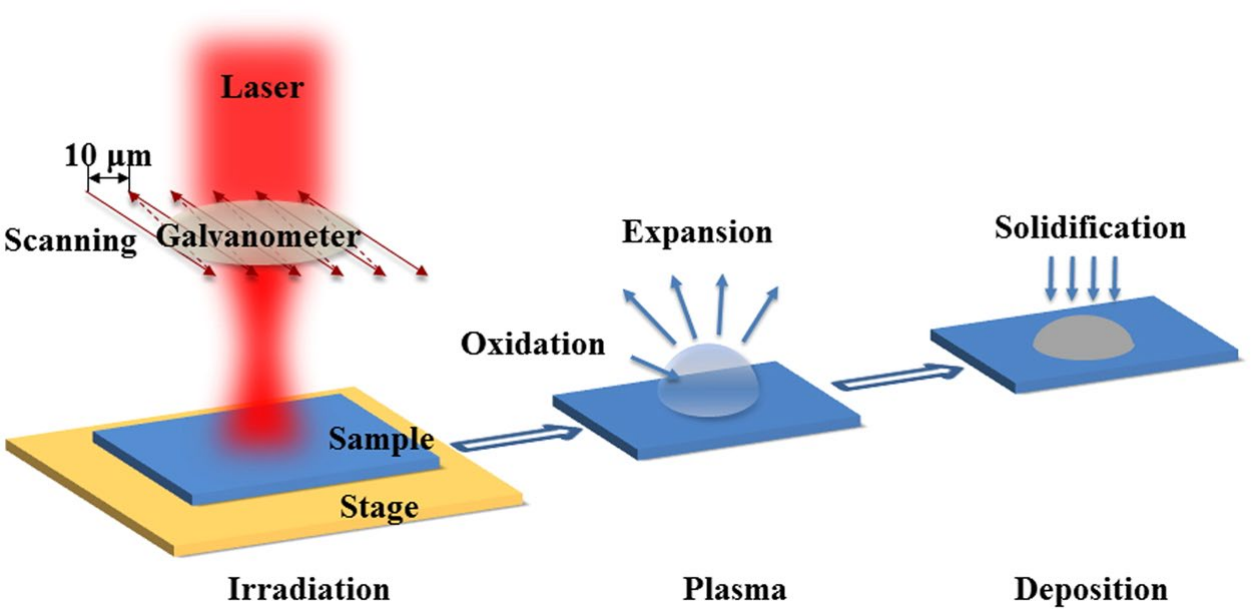
Schematic illustration of the irradiation scanning process of SiC.

The 6H-SiC samples were modified using a picosecond pulsed laser. A laser-irradiation scanning process in air was designed, as illustrated in Fig. 1. The size of laser spot was about 100 μm under +2.8 mm defocusing during irradiation. Scanning was achieved by a galvanometer. The repetition rate, distance between scanning paths and scanning speed were adjusted to modulate the temporal and geometric intervals between laser pulses to accomplish uniform and large-scale pattern fabrication. The power intensity of the laser determined the output of this process. Hemispheres of deposited SiC were observed using a power intensity of 40–80 MW/cm^2^. Higher power intensity resulted in material ablation but not the formation of hemispheres based on the balance between the expansion and deposition of the plasma.

**Figure 2:**
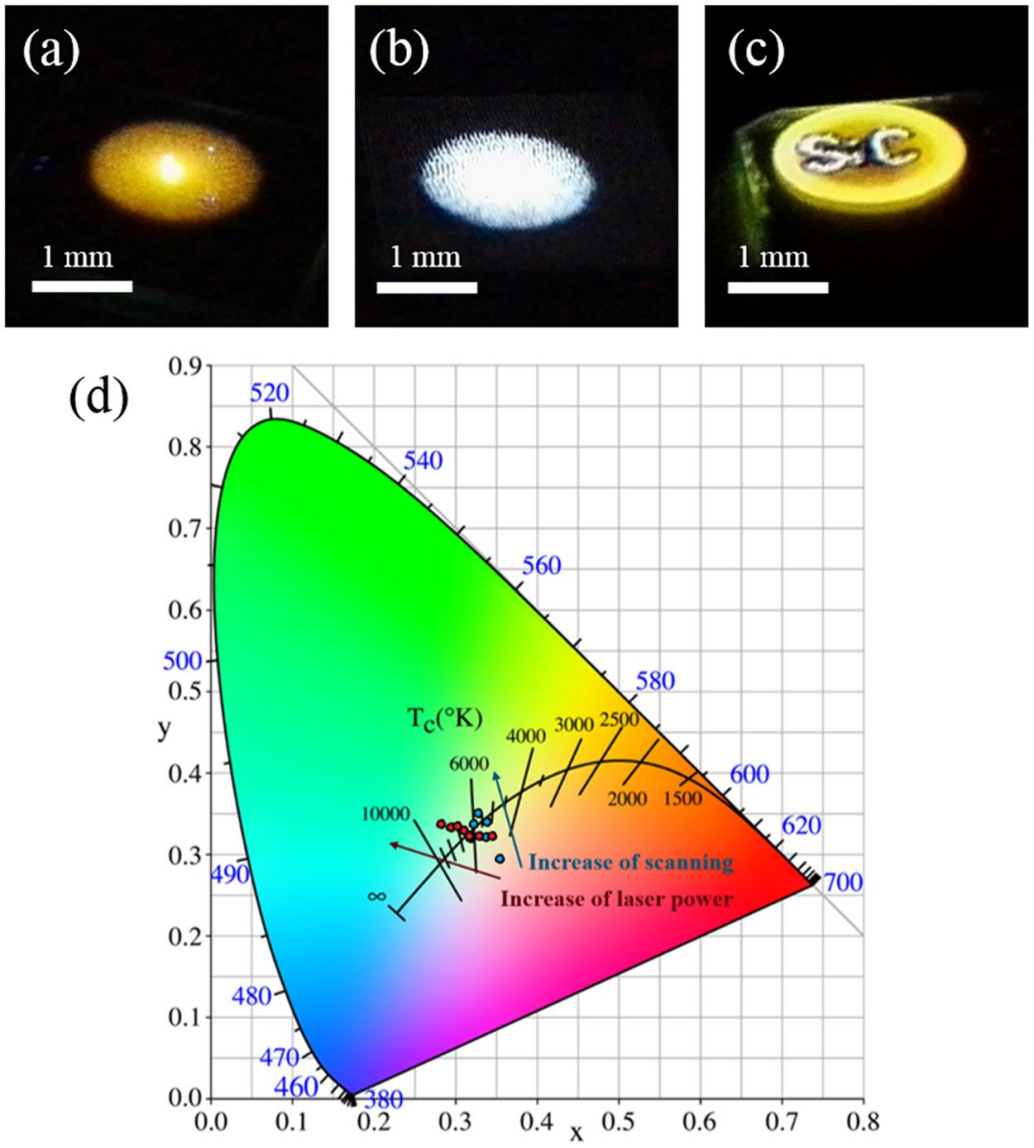
Photographs of the light emission of a (**a**) pristine 6H-SiC surface, (**b**) laser-irradiated 6H-SiC surface (white-PL sample), and (**c**) laser-patterned “SiC” on a 6H-SiC surface following excitation by a 325-nm He–Cd laser. (**d**) The 1931 CIE chromaticity diagram of samples fabricated with different parameters. The red dots from right to left represent the emission of the samples using increasing laser power and the blue dots represent the emission of samples scanned by different scanning numbers.

The luminescence of the untreated n-type 6H-SiC exhibited yellowish orange under UV laser excitation, as shown in Fig. 2(a,d). This emission was displayed following excitation by a defocused He-Cd laser (325 nm) at room temperature. The spot diameter was ~2 mm. Figure 2(b) shows the emission of laser-treated 6H-SiC under the same excitation conditions. The scanning process allowed us to fabricate arbitrary patterns on the SiC wafer. Figure 2(c) shows an example of a pattern: the characters “SiC” scanned by the laser exhibited white luminescence, while the SiC background exhibited the original yellow luminescence. By increasing the laser power intensity from 40 to 80 MW/cm^2^, the PL of the treated surface turned from yellowish white to pure white to bluish white, the emission colour shifted to bluish white and the colour temperature quickly increased from 4193 K to a maximum of 8224 K. Similarly, the colour temperature increased as the number of repeated laser scans increased. These tendencies can be observed on the chromaticity diagram in Fig. 2(d). The bluish emission with a high colour temperature is not desired, so the excess blue component needs to be eliminated. Although the maximum PL intensity was obtained at 75 MW/cm^2^ and 300 scans, the operating window of laser power intensity should be around 65 MW/cm^2^ to achieve a desirable colour temperature. The calculated chromaticity coordinates of the white emission were *x* = 0.3385 and *y* = 0.3400 in the 1931 CIE chromaticity diagram, and the colour temperature was 5227 K. The emission of the laser-treated samples was similar to pure white sunlight (*x* = 0.33, *y* = 0.33 and colour temperature of 5500 K). We denoted this sample as the white-PL sample.

**Figure 3:**
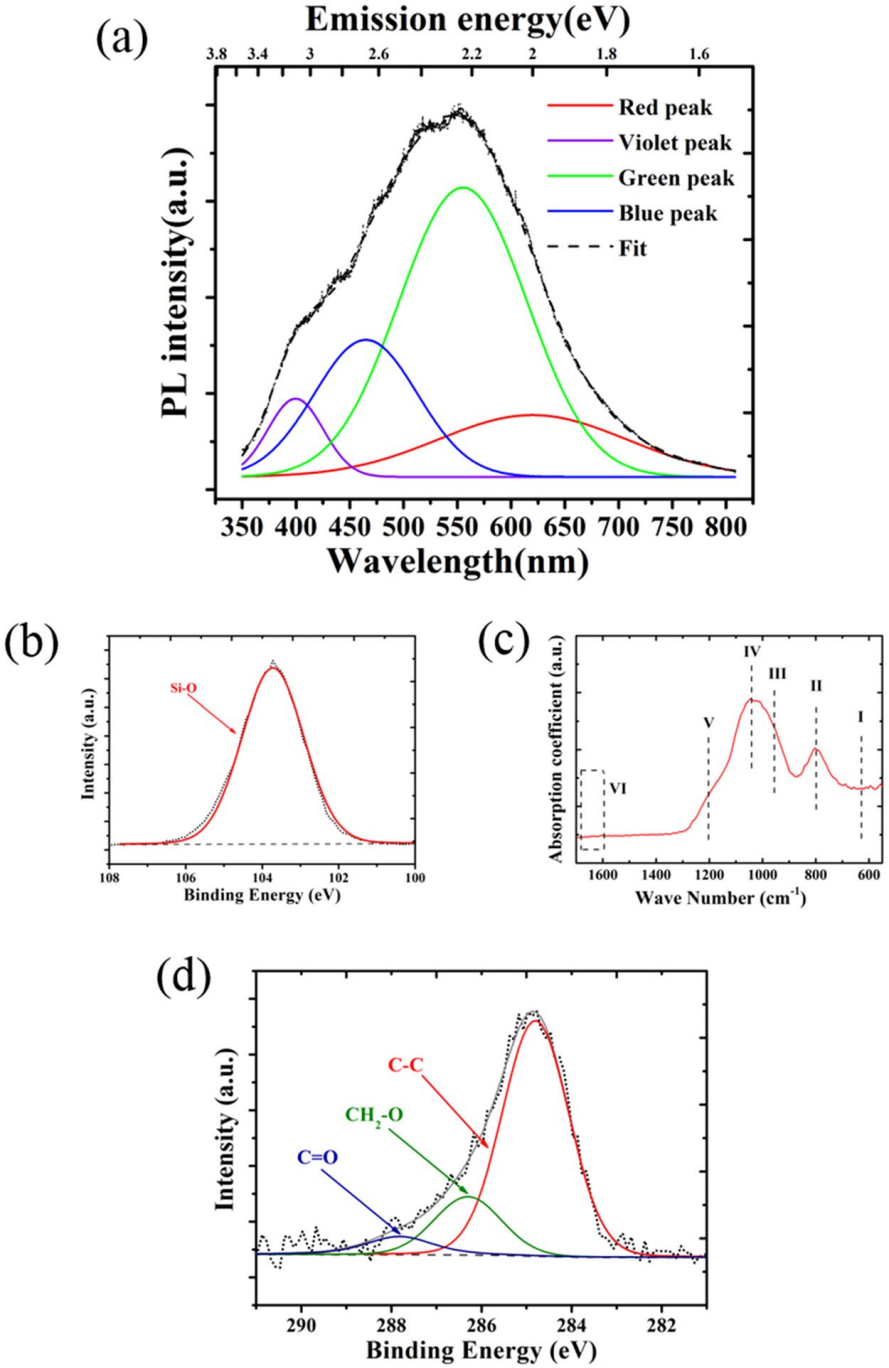
(**a**) PL spectrum of the laser-irradiated white-PL sample. The spectrum is deconvoluted into four PL bands. XPS analysis of the white-PL sample, showing the (**b**) Si 2p and (**c**) C 1 s spectra. (**d**) FTIR spectrum of the white-PL sample.

The PL emission of the white-PL sample under 325-nm laser excitation covers the full spectrum in the visible wavelength range from 350 to 800 nm with maximum intensity at 550 nm, as shown in Fig. 3(a). The PL emission spectrum can be deconvoluted into four emission peaks, centred at ~ 400 nm (violet peak), ~ 459 nm (blue peak), ~ 563 nm (green peak) and ~620 nm (red peak). According to previous reports^14–18^, the non-bridging oxygen hole centre (NBOHC; $\equiv {\rm{Si}}-{\rm{O}}\cdot$) with an absorption band at 258 nm (4.8 eV) shows red PL at 620–653 nm (1.9–2.0 eV); silanone $(={\rm{Si}}={\rm{O}})$ displays a green PL band at 546 nm (2.27 eV); the Si-related neutral oxygen vacancy (NOV; $\equiv {\rm{Si}}-{\rm{Si}}\equiv$) shows a blue PL band at 442–459 nm (2.7–2.8 eV); and two-coordinate silicon (silylene; $={\rm{Si}}:$) is the origin of the violet PL band at 400 nm (3.1 eV). In addition, computational calculations indicated that dioxasilyrane $(={\rm{Si}}({{\rm{O}}}_{2}))$, the two oxygen molecules are bonded by a weak ${\rm{O}}-{\rm{O}}$ bond) exhibits PL bands from 539 nm (2.3 eV) to 605 nm (2.05 eV) and from 689 nm (1.8 eV) to 729 nm (1.7 eV)^16^.

The bonding structure and chemical composition of the white-PL samples were analysed using X-ray photoelectron spectroscopy (XPS). The contents of Si, C and O in the sample were found to be 27.56%, 4.78% and 67.26%. Thus, the dominant phase was a SiO_2_-like phase with excess oxygen. This nonstoichiometric result suggested that the material contained numerous oxygen-rich defects. Figure 3(b,c) show the Si 2p and C 1 s spectra of the white-PL sample. The only peak at 103.8 eV in the Si 2p spectrum was assigned to Si-O bonds. Without Si-C (100.8 eV), the Si-O bonds were the dominant silicon bonds on the surface, which revealed that the irradiated layer was composed of Si-O bonds. The deconvoluted C 1 s spectrum in Fig. 3(c); without the C - Si bonds (282.9 eV), the main carbon bonds were C-C (284.8 eV), CH_2_-O (286.3 eV) and C=O (287.2 eV)^19,20^. Our previous work using a nanosecond pulsed excimer laser (~10^7^ W/cm^2^, 248 nm, 5.0 eV) demonstrated that the enhancement of blue PL of SiC originated from shallow defects induced by nitrogen implantation^21^. The different behaviour observed here indicates that nitrogen was not involved in this infrared (IR) picosecond laser (1064 nm, 1.2 eV) treatment. The ionisation of the gas molecules is wavelength-dependent because the photon energy and power intensity are too low to cause the cleavage of nitrogen^22^.

A Fourier transform infrared (FTIR) spectrum of the white-PL sample is displayed in Fig. 3(d). Various silicon–oxygen-related absorption bands were observed in the range from 700 to 1500 cm^−1^. The absorption intensity at about 620 cm^−1^ (I) is assigned to the stretching vibration of ${\rm{O}}-{\rm{O}}$ weak bonds. The first peak around 800 cm^−1^ (II) is assigned to the Si-O bending motion in SiO_2_. The most intense region at approximately 850–1300 cm^−1^ is dominated by three asymmetric peaks centred at about 950 cm^−1^ (III), 1050 cm^−1^ (IV) and 1180 cm^−1^ (V) consistent with the stretching vibration of Si-OH groups, Si-O-Si stretching mode and Si-O asymmetric stretching mode, respectively^17,23–25^. C=C bonds at about 1620~1680 cm^−1^ was absent, which would result from the sp^2^-carbon molecular orbitals^26^. The dominant carbon bond was C-C indicated by the XPS results. It was concluded that elemental carbon in the form of the diamond phase was dispersed in the Si-O-Si system. The above results indicate that the main component of the surface layer following irradiation was silicon oxycarbide (SiC_x_O_y_).

**Figure 4:**
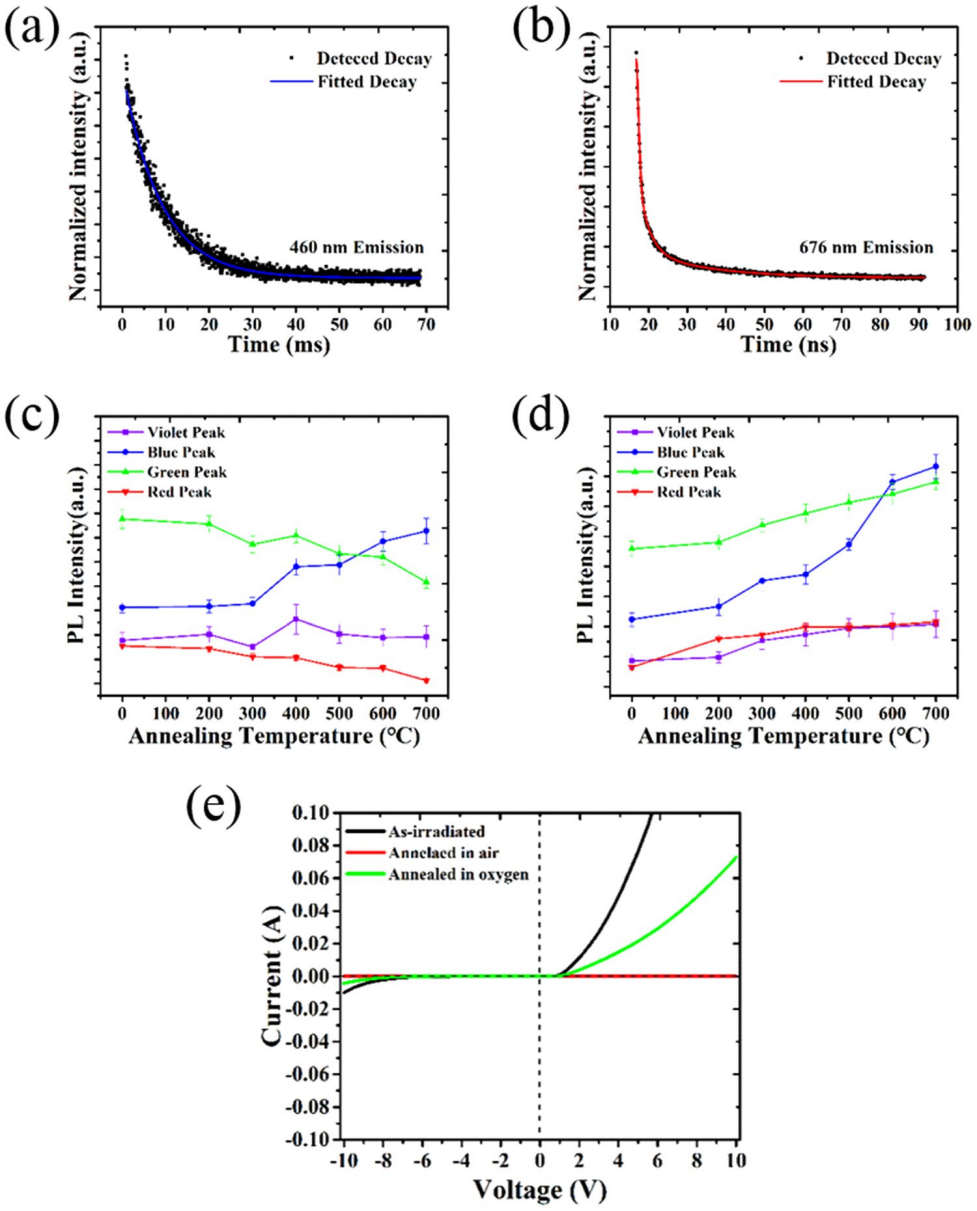
Fitted decay traces of emission at (**a**) 460 nm and (**b**) 676 nm of the white-PL sample. Intensities of the four deconvoluted peaks of samples annealed in (**c**) air and (**d**) oxygen at different temperatures. (**e**) Current–voltage curves of the white-PL sample and samples annealed in air and oxygen atmosphere.

To further explore the assignment of the PL band originating from defects, the time-resolved PL features were investigated and annealing experiments were conducted in air and oxygen atmosphere. Two different lifetime characteristics of the PL decay were found for the 460 nm (2.7 eV) and 676 nm (1.8 eV) emission bands, as shown in Fig. 4(a,b). Monoexponential decay kinetics on the millisecond scale were observed for the slow decay. The derived PL lifetime of the 2.7 eV emission is 8.83 ms, representing the triplet-to-singlet transitions of NOVs^15^. The decay trace of the fast band fitted a multiple exponential decay function with derived lifetimes of 0.31, 2.68 and 18.00 ns. These results are in good agreement with the experimental values for triplet-to-singlet and singlet-to-singlet transitions of silica defects^16,18^. The PL lifetime of NBOHCs is believed to be 10–20 μs. Although decay on the microsecond scale was not observed here, the existence of NBOHCs cannot be excluded because of the absorption band observed at 4.8 eV. We were able to assign the PL components to the Si-related oxygen defects as follows: the red PL (1.8–2.0 eV) is caused by NBOHCs and dioxasilyrane; the green PL (2.2 eV) is related to silanone groups; the blue PL (2.7 eV) originates from NOVs; and the violet PL (3.1 eV) is caused by silylene groups. Because the concentration of C was under 5%, the PL behaviour of C components is not discussed in the present work. The combination of the four bands resulted in full-spectrum light emission. The four PL components were assigned to specific defects, in good agreement with the Gaussian fitting of four PL peaks in Fig. 3(a). Considering the coordination number of a silicon atom, NBOHCs and dioxasilyrane groups are oxygen-excess centres (OECs), showing p-type behaviour in the SiO_2_ matrix, whereas NOVs and silylene groups are oxygen-deficient centres (ODCs) and show n-type behaviour.

Figure 4(c,d) display the maximum PL intensities of samples annealed by irradiation with a continuous-wave (CW) IR laser in air and oxygen. The most remarkable difference between the samples was the different tendencies of the PL peaks as annealing temperature increased. When annealed in air above 400 °C, the intensity of red and green emission peaks decreased as the temperature rose, while that of the blue band increased and that of the violet fluctuated slightly. The air acted as an oxygen-deficient environment during the annealing process. As described by the derived reactions between defects and oxygen (Equations (1) and (2), where (*g*) means gas), low oxygen partial pressure leads to more ODCs and less OECs. When annealing began, the oxygen-excess bonds gave up the oxygen to compensate the lack of Si coordination number, causing the intensity of the corresponding PL bands to decrease. The same annealing experiments performed in oxygen showed an overall increase in the intensities of all four peaks. It is clear that oxygen was involved in the annealing process. The abnormal intensity increase of blue and violet peaks indicated that under high oxygen partial pressure, reaction (3) is dominant between the defects and oxygen.

1 $${\rm{ODC}}+{{\rm{O}}}_{2}({\rm{g}})\leftrightarrow {{\rm{SiO}}}_{2}$$

2 $${\rm{OEC}}\leftrightarrow {{\rm{SiO}}}_{2}+{{\rm{O}}}_{2}({\rm{g}})$$

3 $${{\rm{SiO}}}_{2}+{{\rm{O}}}_{2}({\rm{g}})\leftrightarrow {\rm{ODC}}+{\rm{OEC}}$$

Simple electrical tests of the white-PL and annealed samples were performed to explore the properties of the luminescent centres. The rectification behaviour of the p-n junction formed using the white-PL sample is shown in Fig. 4(e). The target material n-type 6H-SiC remained as the provider of n-type carriers. NBOHCs behaved as p-type carriers because the oxygen-dangling bond could trap an electron to form a hole. After annealing in air, the p-n junction was inactivated because the density of OECs decreased after compensating the lack of Si coordination number. However, the SiC_x_O_y_ annealed in oxygen retained its p-type properties. The conductivity of the samples being affected by the annealing process could be explained in two ways: first, the activation and compensation of OECs occurs at the same time during oxygen annealing; second, thermal activation of ODCs leads to the increase of n-type carrier concentration and decrease of p-type carrier concentration.

**Figure 5:**
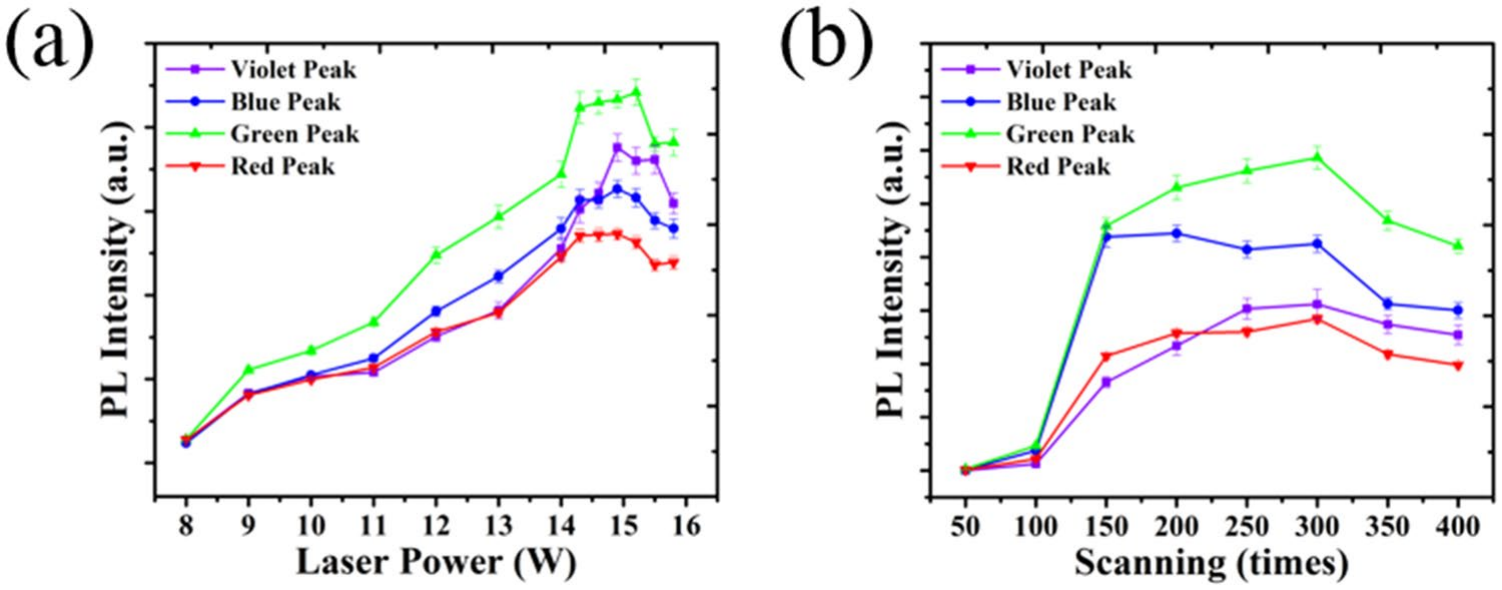
Intensity dependence of the four deconvoluted peaks on (**a**) the laser power intensity and (**b**) the number of scans.

The factors that affect the luminescent properties of the samples were investigated by adjusting the laser parameters of laser power and scanning number in the surface-modifying process. The defect-generating reactions were thus controllable, allowing high-quality white or tuneable emission to be realised. The relative intensities of the four fitted peaks were used to estimate the effects of different laser parameters on the emission spectra. First, samples were irradiated 300 scans with a laser power from 40 to 80 MW/cm^2^. The four PL peaks of these samples are compared in Fig. 5(a). Up to a laser power of 70 MW/cm^2^, the intensities of three peaks increased with laser power. At around 75 MW/cm^2^, the intensities of all four emission bands simultaneously reached their maximum values and then decreased. The PL decrease at higher laser power was caused by the sputtering of the plasma components.

The spectra in Fig. 5(b) demonstrate the peak behaviour as the number of scans increased. The PL intensity of all peaks increased markedly and the emission became bluish white when the number of pulses reached 150. The maximum PL intensities of red, green and violet bands were obtained after 300 scans, while the change of the blue band was not obvious. We noticed that when the number of scans was changed from 250 to 300, the intensity increases of red and green bands were greater than those of blue and violet bands, which indicates that at this stage, the coordination number of Si could not decrease further but the formation of oxygen vacancies and oxygen dangling bonds still continued. The decrease of PL intensity observed for samples sputtered more than 300 scans can be explained by the sputtering of the material.

The vapourisation temperature of SiC is 3300 K according to the Si-C phase diagram established by Kleykamp and Schumacher^27^. The calculation based on one-dimensional heat conduction model^28–32^ indicated that the surface temperature of SiC can reach the vapourisation point when the laser power intensity is 40 MW/cm^2^. Plasma is formed during the sublimation when the laser fluence is higher than the breakdown threshold of a material^33^. Because of the ability of the picosecond laser to release a large amount of energy in a short time, the laser pulse will excite electrons through multiphoton and impact ionisations and emit electrons from the surface and underlying regions, inducing strong ionisation and leaving a high concentration of uncompensated ions.

At an average laser power of 75 MW/cm^2^, the highest temperature reached 5000 K, which is much higher than the vapourisation and oxidation temperatures of SiC^27,34^. The following vapour–solid reaction mechanism^35^ was proposed to describe the interactions: the SiC substrate is preferentially oxidised by O_2_ (Equation (4)), the cooling and nucleation of SiO_2_ (Equation (5), where (*s*) means solid) and SiO_2_ is converted into SiO (vapour) and then SiO is oxidised into SiO_2_ (Equation (6)). In addition, the kinetics of the melt–regrowth process in this material is not accessible because of the lack of any valuable information related to the properties of molten SiC. Therefore, the simulations were restricted to determining the irradiation conditions required to heat the surface of SiC to its vapourisation temperature.

4 $${\rm{SiC}}({\rm{g}})+{{\rm{O}}}_{2}({\rm{g}})\to {{\rm{SiO}}}_{2}({\rm{g}})+{{\rm{CO}}}_{2}({\rm{g}})$$

5 $${{\rm{SiO}}}_{2}({\rm{g}})\to {{\rm{SiO}}}_{2}({\rm{s}})$$

6 $${{\rm{SiO}}}_{2}\leftrightarrow {\rm{SiO}}({\rm{g}})+{{\rm{O}}}_{2}({\rm{g}})$$

SiC_x_O_y_ materials are a promising platform to realise direct white light emission because of their potential for desirable chromaticity and wide tuneable optical bandgaps^14,26,36–38^. Compared with other techniques to prepare SiC_x_O_y_ materials such as the sol–gel method^39,40^, C-ion implanted SiO_2_^41^, chemical vapour deposition^42,43^ and direct-current arc discharge plasma^38^, the advantage of laser irradiation method is in the efficiency of the process. Using our method, large areas of patterned surface structure can be fabricated in one step. This process involving vapourisation, oxidation and deposition may be adapted to other kind of materials useful for light emission because of the universal nature of laser–material interactions. Regarding stability, after being stored at room condition for 90 days, the variation of PL intensity of the samples was within 5%. Also, there was no marked change of PL intensity observed when the samples were heated up to 200 °C. It is worth noting that for the samples annealed in air, the intensity of the blue component enhanced while those of the green and red components weakened as the annealing temperature rose, leading to the increase of colour temperature from ~5500 to ~8000 K. In this case, using an oxygen atmosphere during annealing is crucial because of its ability to enhance the intensities of green and red PL components; as a result, the excessive increase of colour temperature is limited (up to ~6200 K).

## Conclusion

In summary, full-spectrum white PL was demonstrated from ps laser irradiated SiC under UV excitation. The treated surface was tailored from yellowish white to pure white to bluish white, and the emission colour shifted to bluish white and colour temperature quickly increased from 4193 K to a maximum of 8224 K upon modulating laser irradiation conditions. The optimised laser power intensity of 65 MW/cm^2^ achieved a desirable colour temperature similar to that of sunlight (x = 0.33, y = 0.33 and colour temperature of 5500 K) and suppressed blue emission. Oxygen-related defects that acted as luminescent centres were generated by oxidation during the vapour–solid process induced by ps laser irradiation. The laser scanning process showed high controllability and adaptability in tailoring the relative intensities of PL bands. Annealing experiments were performed to demonstrate the nature of the luminescent centres on the SiC surface. In addition, p-n junctions were formed using n-type SiC as the target material. Optimising the structure of the devices should lead to further improvements in performance and eventually electronically driven white light from a single diode.

## Methods

### Materials and experimental setup

The starting materials were (0001)-oriented single-crystal epitaxy-ready SiC wafers (TankeBlue, China) with both sides polished. The samples were 6 H polytype and heavily doped with nitrogen (10^18^ cm^−3^) with a resistivity of 0.1 Ω•cm. Wafers were mechanically cut into 5 × 5 mm samples with a thickness of 340 μm.

The laser in this work was a picosecond pulse laser (Edgewave, Germany) with an IR laser scanning system with a wavelength of 1064 nm and pulse duration of 10 ps. The focal length was 100 mm. The focal spot size on the workpiece surface was 100 μm under defocusing conditions. The average power of the laser was from 8 to 16 W, which gave a power intensity of 40–80 MW/cm^2^ based on the threshold of the SiC vapourisation temperature. The repetition rate of the laser is 200–500 kHz. To fabricate a large-scale modified surface and arbitrary patterns, the laser scanning was performed in air 0–400 passes on the same plane within one scanning path. The interval between scanning paths was 10 μm. Laser scanning was realised by a laser galvanometer with a maximum scanning speed of 3000 mm/s, and the focal point of the laser beam could be adjusted.

### Laser annealing

Annealing experiments were carried out using a commercial IR fibre continuous wave (CW) laser (1064 nm) with a maximum power of 80 W. The laser spot was set to 10 × 10 mm. During annealing, the temperature of samples was monitored by an IR thermometer, and the power dependence of temperature was almost linear, which could be given by ${T}_{anneal}=361+8\ast {P}_{laser}$ (K). The samples were prepared in advance with the picosecond laser using the following parameters: laser power of 13 W, the number of scans of 300 and defocusing distance of +2.8 mm. The samples were first annealed for different times, from which we found that 5 min was the optimum annealing period. Samples were then irradiated in air and pure oxygen at atmospheric pressure. Annealing temperatures of 200, 300, 400, 500, 600 and 700 °C were used.

### Characterisation

PL spectra were obtained using a CW He-Cd laser (325 nm) as the excitation source at room temperature in air. The laser power was approximately 9 mW. Time-resolved PL emission spectra, CIE 1931 chromaticity diagrams and excitation spectra were acquired on a spectrometer (Edinburgh Instruments, FLS980) equipped with an ozone-free xenon arc lamp and nanosecond flash lamp. XRD patterns were recorded on a Bruker D8 diffractometer. XPS measurements were carried out on an Axis Ultra spectrometer to identify the elemental distribution and bonding states of samples. FTIR spectra were measured on a Nicolet Magna-IR 750 FTIR spectrometer to record the local atomic environment and bonding configuration of samples. Scanning electron microscopy (SEM; JEOL, JSM 6500 F) and laser scanning confocal microscopy (LEXT, OLS3100) were used to characterise the surface structure of samples.

Test devices obtained by coating In–Ga alloy electrodes on both sides of the samples were used to study the electronic properties of annealed and unannealed SiC_x_O_y_. The test device structure was In–Ga alloy/SiC_x_O_y_/n-6H-SiC/In–Ga alloy. Before coating, the surfaces of the samples were irradiated by a KrF excimer laser (248 nm) to improve the ohmic contact between electrodes and SiC. Current–voltage curves were obtained with an electronic property testing system.

## Electronic supplementary material

Supplementary Information
