# Author Correction: Selective 6H-SiC White Light Emission by Picosecond Laser Direct Writing

**DOI:** 10.1038/s41598-018-24101-y

**Published:** 2018-04-19

**Authors:** Sicong Wang, Lingfei Ji, Lin Li, Yan Wu, Yongzhe Zhang, Zhenyuan Lin

**Affiliations:** 1 Institute of Laser Engineering, Beijing University of Technology, Beijing, 100124 China; 2 Laser Processing Research Centre, School of Mechanical, Aerospace and Civil Engineering, The University of Manchester, Manchester, M13 9PL UK; 3 College of Material Science and Engineering, Beijing University of Technology, Beijing, 100124 China

Correction to: *Scientific Reports* 10.1038/s41598-017-18685-0, published online 10 January 2018

This Article contains an error in the Acknowledgements section.

“This work was supported by the National Science Foundation of China under grant no. 50875006. The authors thank the technical support from Professor Pingheng Tan.”

should read:

“This work was supported by the National Science Foundation of China under grant no. 51575013. The authors thank the technical support from Professor Pingheng Tan.”

