# Supplementary material for: Selective 6H-SiC White Light Emission by Picosecond Laser Direct Writing
Source: Sci Rep. 2018 Jan 10;8:257. doi: 10.1038/s41598-017-18685-0 (PMC5762700; doi:10.1038/s41598-017-18685-0)
Supplement: Supplementary file 1 — Supplementary Information [file 41598_2017_18685_MOESM1_ESM.pdf]

Supplementary Information

Title

Selective 6H-SiC White Light Emission by Picosecond Laser Direct Writing

Authors

Sicong Wang[1], Lingfei Ji[1], Lin Li[2,1] , Yan Wu[1], Yongzhe Zhang[3], Zhenyuan Lin[1],
[1]Institute of Laser Engineering, Beijing University of Technology, Beijing, 100124, China
[2] Laser Processing Research Centre, School of Mechanical, Aerospace and Civil Engineering, The University of Manchester, Manchester, M13 9PL, UK
[3] College of Material Science and Engineering, Beijing University of Technology, Beijing, 100124, China
Correspondence: Professor LF Ji, Institute of Laser Engineering, Beijing University of Technology, Beijing, 100124, China
Professor L Li, Laser Processing Research Centre, School of Mechanical, Aerospace and Civil Engineering, The University of Manchester, M13 9PL, UK
Email: ncltji@bjut.edu.cn
lin.li@manchester.ac.uk

Information

The complex particles[1] generated in the vapor plume make it hard to define the accurate reaction. Hence, we discuss the origin and effects of the PL shift by the condition of products. Generally, a blue shift of PL can be induced by decreasing crystal size or changing material composition, or both. Nanoparticles with a size of ~100 nm were observed in an SEM image of the surface of the white-PL sample (Figure S1). However, to efficiently influence the band gap of semiconductor materials through the quantum confinement effect, the size of the crystal has to be decreased to the order of the Bohr radius, i.e., 2.7 nm for 3C-SiC[2], to affect its absorption properties and cause a blue shift of light emission. These particles with a size of ~100 nm have a high surface-to-volume ratio, which raises the intensity of surface states, but hardly display the quantum confinement effect. The results of XRD measurement of the sample are illustrated in the inset of Figure S1. In addition to the strongest diffraction peak at 2θ = 35.6° originating from

α-SiC (111) of the 6H-SiC single-crystalline substrate, an amorphous halo centred at 2θ = 22°–24° with a full width at half maximum of 10.7° can be observed, which indicates the presence of amorphous SiO_x structure in the laser-treated SiC[3].

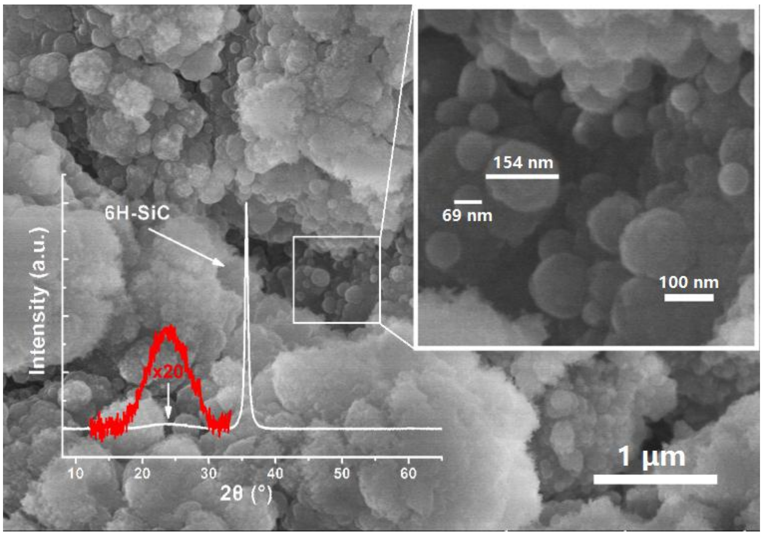

Figure S1 SEM image of the white-PL SiC_xO_y sample (particle size of ~100 nm). Inset is the XRD pattern of the sample. The peak at 2θ = 35.6° is characteristic of 6H-SiC; the silica halo in red is magnified by 20 times.

Excitation–emission matrix spectroscopy was conducted with an excitation wavelength range from 240 to 340 nm and emission wavelength range from 300 to 700 nm, as shown in Figure S2. The main emission was observed at 460 nm (2.7 eV) following excitation at photon energy higher than 4.8 eV (~ 260 nm). When the excitation energy was lower than 4.4 eV, a wide band ranging from 550 to 680 nm emerged, covering the emission photon energy range from 1.8 to 2.3 eV. Two obvious sharp peaks in this range were found at 615 nm (2.0 eV) and 676 nm (1.8 eV), corresponding to two radiative transitions of dioxasilyrane.

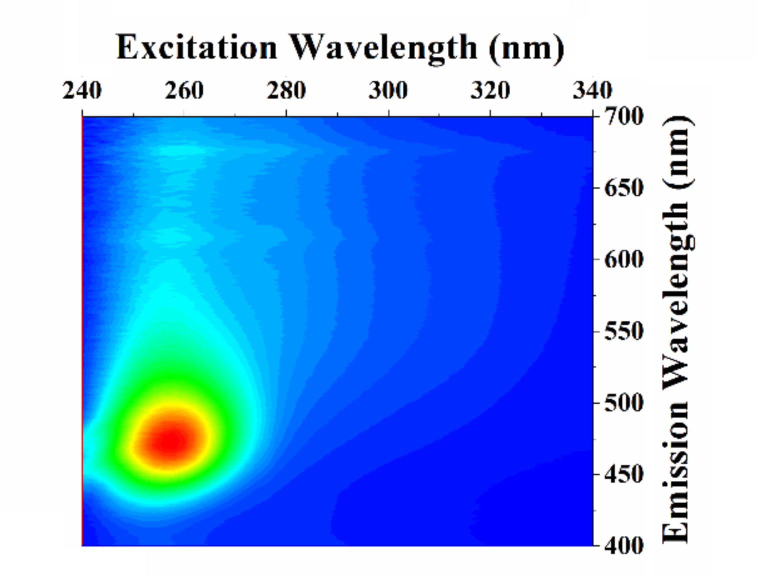

Figure S2 Excitation–emission matrix spectroscopy (contour map) of the white-PL sample.

Temperature calculation

The basic mechanism of laser–solid interaction involves heat generation on the surface of the irradiated area upon light absorption, followed by cooling through heat conductivity into the substrate. Heating and cooling stages can be determined by numerically solving the heat flow equation[4-6],

$$C\rho\frac{\partial T(x,t)}{\partial x} = \frac{\partial}{\partial x}\left(k\frac{\partial T(x,t)}{\partial x}\right) + S(x,t), \quad (1)$$

where C represents the specific heat capacity (J/g K), ρ the density (g/cm^3) and k the thermal conductivity (W/cm K) of the irradiated material. Temperature T in Kelvins and heat generation S are functions of space and time. S is described as,

$$S\,(x,t) = P(t)\alpha(1-R)\exp(-\alpha x), \quad (2)$$

and depends on the optical absorption α and surface reflectivity R of the material at the IR wavelength. $P(t)$ is defined as the time-dependent power density of the laser beam, which is assumed to be uniform in space and with a Gaussian distribution in time. The sublimation temperature of SiC is 3300 K according to the Si-C phase diagram established by Kleykamp and Schumacher[7]. The optical properties were deduced from reflectivity and spectroscopic ellipsometry measurements[8]. The k value of SiC used in our calculations was $4517 - T^{-1.29}$ (W/cm K), as reported previously.[9]

Figure S3 shows the calculated time-dependent surface temperature evolution under laser fluence of 40, 75 and 80 MW/cm^2. The maximum surface temperature generated at a laser fluence of 40 MW/cm^2 is 3100 K, which is nearly the vapourisation point of SiC. This calculation result agrees well with the experimental findings. The irradiated surface became rough and black when the laser power reached 8 W (40 MW/cm2), but no PL was observed under the same excitation conditions. By increasing the laser power from 8 to 16 W, the PL of the treated surface progressed from yellowish white to pure white to bluish white.

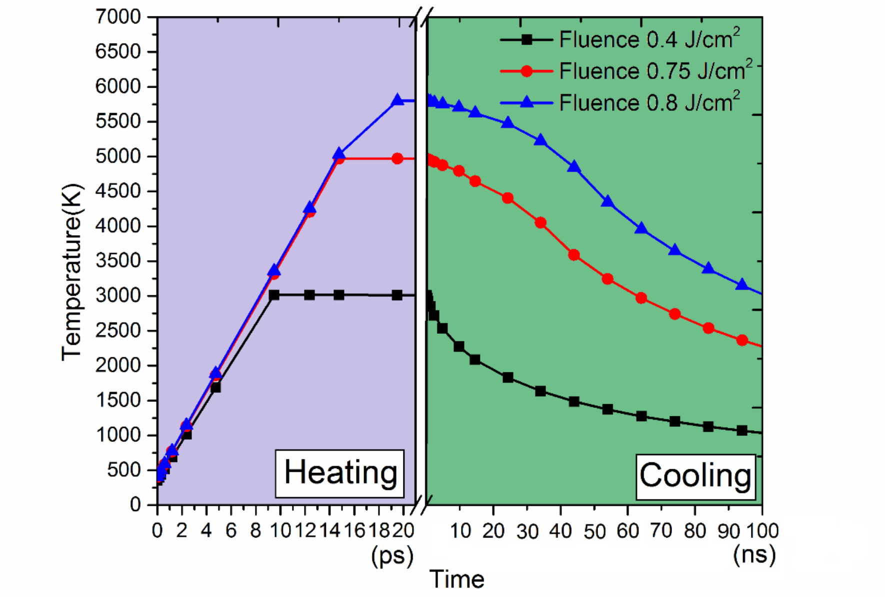

Figure S3 Calculated SiC surface temperature variation at different laser fluence under 10-ps laser pulses at 1064 nm.

Table S1 Laser parameters and colour temperatures of white emissions.

Laser Power Intensity (MW/cm^2)	Laser Average Power (W)	Number of Laser Scans	Color Temperature (K)
60	12	300	4862
65	13	300	5227
70	14	300	6251
71.5	14.3	300	5666
73	14.6	300	6281
74.5	14.9	300	7499
76	15.2	300	7035
77.5	15.5	300	8224
79	15.8	300	6599
65	13	200	4193
65	13	250	5252
65	13	300	5227
65	13	350	5705
65	13	400	5937

References:

1 Ostrikov, K., Colloquium: Reactive plasmas as a versatile nanofabrication tool. *REV MOD PHYS* **77** 489 (2005).

2 Vörös, M., Deák, P., Frauenheim, T. & Gali, A., The absorption spectrum of hydrogenated silicon carbide nanocrystals from ab initio calculations. *APPL PHYS LETT* **96** 51909 (2010).

3 Peña-Alonso, R., Mariotto, G., Gervais, C., Babonneau, F. & Soraru, G. D., New Insights on the High-Temperature Nanostructure Evolution of SiOC and B-Doped SiBOC Polymer-Derived Glasses. *CHEM MATER* **19** 5694 (2007).

4 Tao, S., Jacobsen, R. L. & Wu, B., Physical mechanisms for picosecond laser ablation of silicon carbide at infrared and ultraviolet wavelengths. *APPL PHYS LETT* **97** 181918 (2010).

5 Leonard, J. P. & Im, J. S., Stochastic modeling of solid nucleation in supercooled liquids. *APPL PHYS LETT* **78** 3454 (2001).

6 Wu, B. & Shin, Y. C., A self-closed thermal model for laser shock peening under the water confinement regime configuration and comparisons to experiments. *J APPL PHYS* **97** 113517 (2005).

7 Kleykamp, H. & Schumacher, G., The Constitution of the Silicon-Carbon System. *Zeitschrift Für Elektrochemie Berichte Der Bun...* (2010).

8 Larruquert, J. I., Pérezmarín, A. P., Garcíacortés, S., Rodríguezde, M. L. & Aznárez, J. A., Self-consistent optical constants of SiC thin films. *Journal of the Optical Society of America A* (2011).

9 C. Dutto, E. FogrARSSY, D. Mathiot, Numerical and experimental analysis of pulsed excimer laser processing of silicon carbide. Applied Surface Science 184, 362-366 (2001).
